# Evaluation of the Dosimetric Feasibility of Hippocampal Sparing Intensity-Modulated Radiotherapy in Patients with Locally Advanced Nasopharyngeal Carcinoma

**DOI:** 10.1371/journal.pone.0090007

**Published:** 2014-02-28

**Authors:** Guang Han, Dong Liu, Hua Gan, Kyle A. Denniston, Sicong Li, Wenyong Tan, Desheng Hu, Weining Zhen, Zhaohua Wang

**Affiliations:** 1 Department of Radiation Oncology, Hubei Cancer Hospital, Wuhan, PR China; 2 Department of Radiation Oncology, University of Nebraska Medical Center, Omaha, Nebraska, United States of America; The University of North Carolina at Chapel Hill, United States of America

## Abstract

**Purpose:**

The objective of this study was to evaluate the dosimetric feasibility of using hippocampus (HPC) sparing intensity-modulated radiotherapy (IMRT) in patients with locally advanced nasopharyngeal carcinoma (NPC).

**Materials/Methods:**

Eight cases of either T3 or T4 NPC were selected for this study. Standard IMRT treatment plans were constructed using the volume and dose constraints for the targets and organs at risk (OAR) per Radiation Therapy Oncology Group (RTOG) 0615 protocol. Experimental plans were constructed using the same criteria, with the addition of the HPC as an OAR. The two dose-volume histograms for each case were compared for the targets and OARs.

**Results:**

All plans achieved the protocol dose criteria. The homogeneity index, conformity index, and coverage index for the planning target volumes (PTVs) were not significantly compromised by the avoidance of the HPC. The doses to all OARs, excluding the HPC, were similar. Both the dose (D_max_, D_2%_, D_40%_, D_mean_, D_median_, D_98%_ and D_min_) and volume (V_5_, V_10_, V_15_, V_20_, V_30_, V_40_ and V_50_) parameters for the HPC were significantly lower in the HPC sparing plans (p<0.05), except for D_min_ (P = 0.06) and V_5_ (P = 0.12).

**Conclusions:**

IMRT for patients with locally advanced NPC exposes the HPC to a significant radiation dose. HPC sparing IMRT planning significantly decreases this dose, with minimal impact on the therapeutic targets and other OARs.

## Introduction

Several clinical studies have suggested that radiation-induced hippocampus (HPC) injury may play a considerable role in the neurocognitive decline of patients after cranial irradiation, particularly deficits in learning, memory, and spatial processing [1], [2]. Recent preclinical studies have demonstrated that a neural stem cell compartment in the HPC is central to the pathogenesis of neurocognitive deficits observed after cranial irradiation [3], [4]. Due to the important role of the HPC in neurocognitive function, clinical trials, such as Radiation Therapy Oncology Group (RTOG) 0933, have been conducted in an effort to decrease the dose to the HPC during cranial irradiation and to mitigate radiation-induced neurocognitive toxicity [5].

Nasopharyngeal cancer (NPC) is an extracranial malignancy that may incur incidental brain irradiation during its treatment. Given the proximity of the nasopharynx to the medial temporal lobes, and the large radiation fields required to effectively treat locally advanced NPC, stage T3 or T4 patients undergoing radiotherapy are likely at higher risk of radiation-induced HPC injury than early-stage patients. NPC is also one of the most common head and neck malignancies in southern China and Southeast Asia. Definitive radiation with or without chemotherapy remains the primary treatment modality for NPC [6]. Deficits in cognitive function have been reported in patients with NPC after radiotherapy. Lee et al [7] found that after a median of 5.5 years after radiation, 16 NPC patients demonstrated poorer memory and social comprehension than a similar group of patients who were evaluated prior to irradiation. Other studies have also shown that irradiation of the HPC is associated with pronounced cognitive impairment in the learning and memory domains in patients receiving radiotherapy for NPC [8], [9].

Intensity modulated radiation therapy (IMRT) enables the delivery of high radiation dose to a target while sparing the surrounding organs at risk (OAR), thus enhancing the therapeutic ratio. It has been accepted as the preferred and most commonly employed radiation technique for NPC. However, the majority of clinical IMRT plans and protocols do not consider the HPC as an OAR. We believe that preservation of the HPC should reduce the risk of late cognitive sequelae associated with the radiation. This study evaluates the effect of identifying the HPC as an OAR in IMRT treatment planning for locally advanced NPC by comparing IMRT plans with and without HPC avoidance objectives.

## Materials and Methods

### Ethics Statement

The Ethics Committees of Hubei Cancer Hospital approved the protocols. Written informed consent was obtained from all patients or from the designated family member when the patient was unable to complete it.

### Cases

Eight patients with stage T3 or T4 NPC were selected for dosimetric planning analyses. Patients with locally advanced disease were selected because the proximity of the primary tumor to the medial temporal lobes may place them at relatively higher risk of radiation-induced HPC injury than patients at early stages [10], [11]. All patients had diagnostic computed tomography (CT) and magnetic resonance (MR) scans. A thermoplastic mask was used for immobilization. A contrast enhanced CT scan with 3-mm slice thickness was used for simulation and radiation planning. Each patient also underwent a T1-weighted, three-dimensional MRI using a 1.5T MR scanner (GE Medical Systems) with a 1.25-mm slice thickness and IV gadolinium. Anatomic contours were delineated on the fused CT and MR image sets using Eclipse version 6.5 (Varian Medical Systems, Palo Alto, CA).

### Target and OAR definition

Target volumes (GTV, CTV, PTV) were delineated per RTOG 0615 treatment protocol [12]. Dose was prescribed to an involved (PTV_70_) and intermediate risk (PTV_59.4_) PTV. PTV_70_ included all areas of disease with a margin and received 70 Gy in 33 fractions of 2.12 Gy and PTV_59.4_ included subclinical areas of disease and received 59.4 Gy in 33 fractions of 1.8 Gy. Critical normal structures identified as OARs were also contoured and expanded according to RTOG 0615, including the brainstem, spinal cord, optic nerves/chiasm, temporal lobes, eyes/lenses, mandible, temporomandibular joints, brachial plexus, oral cavity (excluding PTVs), salivary glands, cochleae, larynx and esophagus.

### HPC contouring

**Figure 1:**
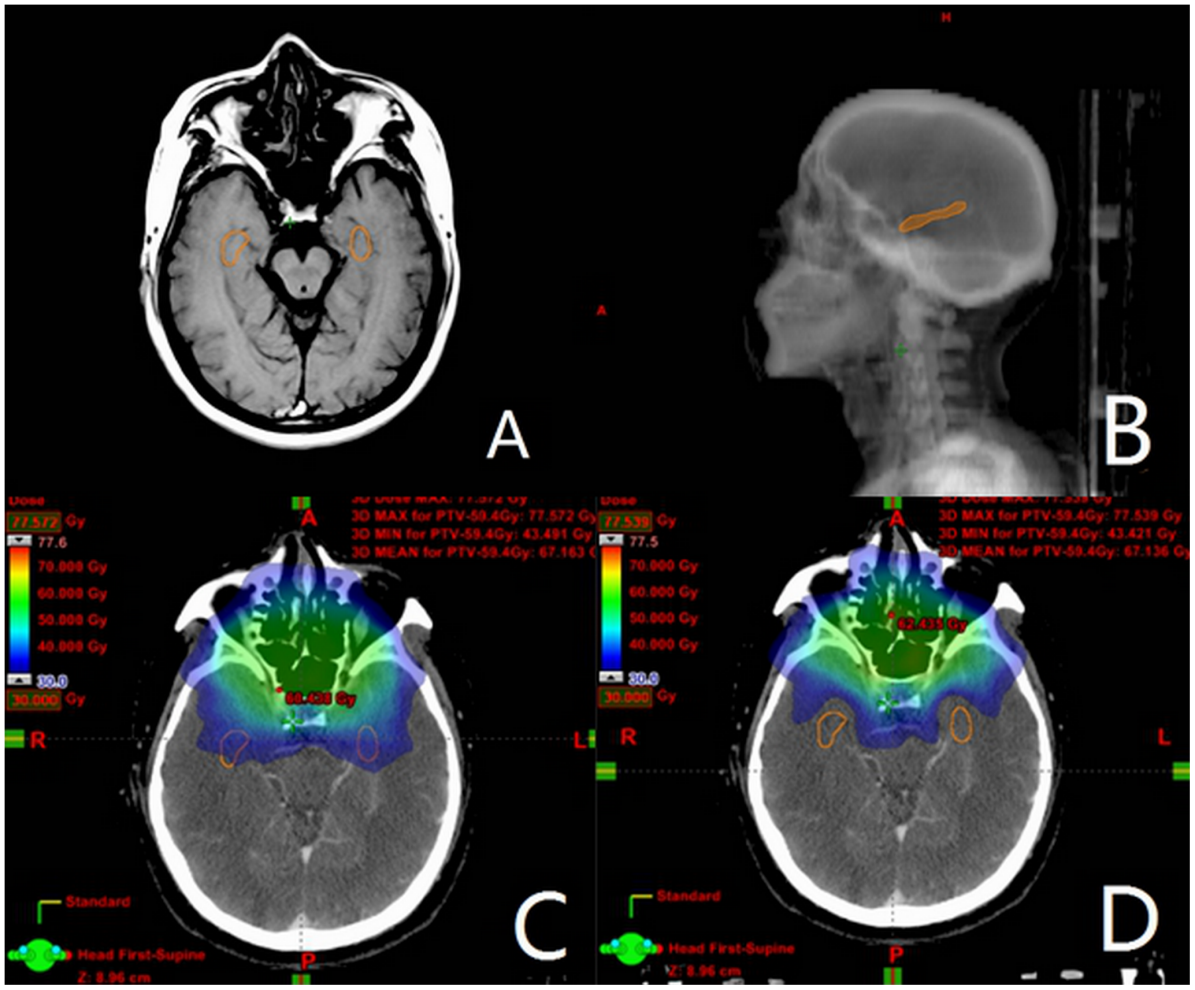
View of dose distribution of the hippocampus contoured on CT-MRI fusion. (A) The hippocampus was contoured on axial T1-weighted MRI. (B) Sagittal DRR image showing hippocampus. (C) Non-HPC sparing IMRT plan. (D) HPC sparing IMRT plan. Orange contour  =  hippocampus. Green dose cloud  = 60 Gy. Light green dose cloud  = 50 Gy. Sky-blue dose cloud  = 40 Gy.

Using the CT-MRI image fusion, the HPC was manually contoured on axial T1-weighted MRI sequences (Figure 1A). The HPC has three anatomic subdivisions: the head, body and tail, and an overall “banana” shape on sagittal images, located in the plane of the lateral ventricle (Figure 1B). According to the method of Gondi et al [13], contouring began at the most caudal extent of the crescent-shaped floor of the temporal horn, continued postero-cranially along the medial edge of the temporal horn, and terminated at the lateral edges of the quadrageminal cisterns, before the emergence of the crus of the fornix. For our study, the bilateral HPC were considered as a single OAR.

### IMRT treatment plans

For consistency, both non-HPC-sparing and HPC sparing plans were developed by the same medical physicist using the standard 9-field co-planar beam IMRT technique. Gantry angles were equally spaced and placed at 0, 40, 80, 120, 160, 200, 240, 280 and 320 degrees, respectively. All plans were to be executed with 6-MV photons using a Varian 23EX linear accelerator. Dose calculation was performed in Eclipse (Varian Medical System) with AAA algorithm using a calculation grid of 2.5 mm [14], and tissue inhomogeneity correction was done using the modified Batho method. The prescribed dose fractionation schedules were derived from RTOG 0615. All plans were normalized in such that at least 95% of the PTV was covered by the prescribed dose. The volume of PTV receiving more than 110% of the prescription dose did not exceed 20%, and that receiving less than 93% of the prescription dose did not exceed 1%. No more than 110% of the prescription dose was outside of the PTV. The dose received by each OAR was limited to the dose constraints according to the RTOG 0615 protocol, with the exception of the HPC. In the HPC sparing IMRT plans, every effort was made to maximally constrain mean dose to the HPC while simultaneously maintaining sufficient PTV coverage and limiting the dose to the other OARs. Dose shaping structures, such as a fan-shaped normal tissue corresponding to a concave target, were utilized during planning. Absolute point doses were measured using a PTW 0.3 cc ion chamber with UNIDOS E dosimeter and the planar dose distributions were measured using IBA MATRIXX ion chamber 2D-Array device.

### Treatment plan evaluation

Dose volume histograms (DVH) were generated for each treatment plan, and the PTV dose coverage and OAR dosimetry were both used for treatment plan evaluation. In addition, the following treatment planning parameters were also considered to evaluate the treatment plans:

Conformity Index (CI) is the ratio of prescription isodose coverage volume to target volume [15]: CI = V_pres_/V_T_, where V_pres_ is the volume receiving a dose greater than or equal to the prescription dose, and V_T_ is the volume of PTV. CI<1.0 indicates that the target volume is not completely covered by the prescription isodose volume and CI>1.0 indicates liberal coverage. A CI value close to 1.0, however, does not imply that the two volumes closely coincide spatially unless the PTV is completely contained within the prescription isodose volume.

Target Coverage (TC) is defined as the ratio of the target volume receiving at least the prescription dose (V_T_, pres) to the entire target volume (V_T_): TC = V_T, pres_/V_T_. If this value equals 1, the target is ideally covered.

Homogeneity Index (HI) indicates dose homogeneity in the target volumes, as recommended by the International Commission on Radiation Units and Measurements [16]. HI is defined as the ratio of the dose difference between the greatest dose delivered to 2% of the target volume (D_2%_) and the dose to 98% of the target volume (D_98%_) to the target median dose (D_median_), HI = (D_2%_−D_98%_)/D_median_. Smaller values of HI correspond to more homogenous target volume irradiation, with a value of 0 indicating absolute homogeneity of dose within the target.

### Plan comparison

The DVH parameters for PTV_70_, PTV_59.4_ and the OARs were calculated. For the PTVs, D_mean_, D_median_, D_min_, D_max_, D_2%_, and D_98%_ were recorded for both the non-HPC sparing and HPC sparing plans. For the OARs, we recorded the D_max_, D_mean_ and D_min_. For the HPC dose analysis, D_mean_, D_40%_, D_median_, D_min_, D_max_, D_2%_ and D_98%_ were calculated, and a set of volumetric parameters, such as V_5_, V_10_, V_15_, V_20_, V_30_, V_40_ and V_50_ were also recorded. The homogeneity index, conformity index, and coverage index for PTV_70_ and PTV_59.4_ were also reported. We then compared the various indices and DVH parameters of HPC sparing and non-sparing plans.

### Statistical analysis

Statistical analysis was performed with the Statistical Package for Social Sciences (SPSS, Chicago, IL) software package, version 17.0, for Windows. All data are expressed as mean ± standard deviation. Paired-sample comparisons were performed, and the paired t test was used. A two-tailed P-Value less than 0.05 was considered as statistical significance in this study.

## Results

### Patient characteristics, PTV and hippocampus volume

As shown in Table 1, the T stage of all eight patients was T3 or T4. The mean volumes for PTV_70_, PTV_59.4_, and the HPC were 424.91 cm^3^ (286.4–532.7), 1054.41 cm^3^ (838.0–1302.6), and 3.15 cm^3^ (2.8–3.5), respectively.

**Table 1:** Tumor stage, PTV and hippocampus volumes.

Cases	TNM stage	PTV_70_ (cm^3^)	PTV_59.4_ * (cm^3^)	Hippocampus (cm^3^)
**1**	T3N0M0	357.2	838.0	3.2
**2**	T4N1M0	430.5	1124.3	3.1
**3**	T3N0M0	290.3	995.6	3.0
**4**	T3N1M0	286.4	899.3	2.9
**5**	T4N2M0	532.7	1210.8	3.3
**6**	T3N1M0	445.8	1080.5	2.8
**7**	T3N1M0	475.6	984.2	3.4
**8**	T4N2M0	580.8	1302.6	3.5
**Mean ±SD**		424.91±107.47	1054.41±156.25	3.15±0.24

Data presented as mean±SD. Abbreviation: T =  tumor; N =  regional lymph node; M =  metastasis; PTV =  planning target volume; SD =  standard deviation.

*PTV59.4 includes PTV70.

### PTV analysis

Ion chamber measured doses were within 3% of calculated doses and the percentage of points passing the gamma criteria (3%, 3 mm) was more than 94.3%. All IMRT plans achieved the protocol dose criteria. Table 2 lists the mean values for DVH parameters of the PTV in IMRT plans with or without HPC sparing. There was no significant difference in D_max_, D_2%_, D_mean_, D_median_, D_98%_ or D_min_ between the HPC sparing and non-sparing plans. As shown in Table 3, the mean values of CI, TC, and HI for PTV_70_ and PTV_59.4_ also showed no significant difference.

**Table 2:** PTV dosimetric parameters with and without HP sparing.

	Parameter	Non-sparing (Gy)	Sparing (Gy)	*P*-value
**PTV_70_**	**D_max_**	77.60±1.44	77.58±1.42	0.68
	**D_2%_**	75.53±0.70	75.48±0.68	0.50
	**D_mean_**	73.36±0.50	73.32±0.53	0.18
	**D_median_**	72.76±0.42	72.75±0.42	0.69
	**D_98%_**	69.59±0.16	69.58±0.18	0.18
	**D_min_**	64.79±1.22	64.68±1.19	0.15
**PTV_59.4_**	**D_max_**	77.30±1.42	77.28±1.43	0.35
	**D_2%_**	76.20±0.63	76.18±0.60	0.45
	**D_mean_**	65.40±0.45	65.40±0.44	0.35
	**D_median_**	64.40±0.37	64.19±0.25	0.18
	**D_98%_**	59.09±0.14	59.03±0.20	0.08
	**D_min_**	51.83±1.33	51.79±1.38	0.20

Data presented as mean±standard deviation. Two-tailed *P* values from paired t tests. Abbreviation: PTV =  planning target volume; D_max_ =  maximum dose; D_mean_ =  mean dose; D_median_ =  median dose; D_min_ =  minimum dose; D_2%_ =  the dose delivered to 2% of the target volume; D_98%_ =  the dose delivered to 98% of the target volume.

**Table 3:** Statistical comparison of HPC sparing and non-HPC sparing IMRT plans.

		Non-sparing	Sparing	*P*- value
**PTV_70_**	**CI**	1.107±0.077	1.132±0.143	0.698
	**TC**	0.969±0.002	0.969±0.002	1.000
	**HI**	0.081±0.011	0.082±0.011	0.196
**PTV_59.4_**	**CI**	1.041±0.079	1.044±0.068	0.876
	**TC**	0.951±0.001	0.952±0.002	0.975
	**HI**	0.266±0.010	0.268±0.011	0.233

Data presented as mean±standard deviation. Two-tailed *P* values from paired t tests. Abbreviation: PTV =  planning target volume; CI =  conformity index; TC =  target coverage; HI =  homogeneity index.

### OAR dose analysis

Table 4 describes the mean D_max_, D_mean_ and D_min_ received by the eyes, lenses, optic nerves, chiasm, brain-stem, spinal cord, cochleae and temporal lobes for the patients using non-HPC sparing plans and HPC sparing plans. In general, OAR doses achieved the protocol dose criteria and were similar between patients. However, compared with non-HPC plans, the HPC sparing plans had decreased dose to some OARs, including the eyes, chiasm and temporal lobes. The maximum dose to the eyes decreased by an average of 7.2%, from 36.38 Gy to 33.75 Gy (p<0.05), and the D_mean_ decreased by 5.0%, from 7.21 Gy to 6.85 Gy (p<0.01). The same trend was observed for the optic chiasm, where the average D_max_, D_mean_, and D_min_ of the non-HPC sparing plans were significantly higher than those for the HPC sparing plans (p<0.001, Table 4). The D_mean_ and D_min_ of the temporal lobes decreased from 20.74 Gy and 1.55 Gy for non-HPC sparing plans to 19.95 Gy and 1.51 Gy in HPC sparing plans (p<0.05).

**Table 4:** D_max_, D_mean_ and D_min_ of various OARs in IMRT plans with and without HPC sparing.

ORA	Parameter	Non-sparing (Gy)	Sparing (Gy)	*P*-value
**Lenses**	D_max_	6.56±0.88	6.49±0.74	0.48
	D_mean_	4.63±0.74	4.64±0.72	0.35
	D_min_	3.55±0.67	3.58±0.64	0.65
**Eyes**	D_max_	36.38±3.67	33.75±4.23	0.03
	D_mean_	7.21±0.84	6.85±0.83	0.00
	D_min_	1.90±0.23	1.91±0.24	0.60
**Temporal lobes**	D_max_	67.83±2.36	67.69±2.43	0.48
	D_mean_	20.74±4.06	19.95±3.97	0.01
	D_min_	1.55±0.38	1.51±0.38	0.04
**Brain-stem**	D_max_	53.06±0.40	53.00±0.44	0.18
	D_mean_	35.59±0.84	35.66±0.85	0.09
	D_min_	9.19±0.30	9.23±0.30	0.44
**Optic Nerves**	D_max_	47.25±1.80	46.60±1.81	0.08
	D_mean_	24.03±4.00	24.29±3.97	0.04
	D_min_	6.51±0.85	6.76±0.93	0.03
**Optic Chiasm**	D_max_	48.15±1.56	46.63±1.59	0.00
	D_mean_	23.61±1.22	21.09±1.09	0.00
	D_min_	9.56±2.75	8.58±2.58	0.00
**Cochleae**	D_max_	58.54±3.62	58.19±3.60	0.06
	D_mean_	42.89±1.53	43.39±1.72	0.01
	D_min_	32.88±2.35	32.94±2.19	0.73
**Spinal Cord**	D_max_	42.76±0.97	42.74±0.93	0.52
	D_mean_	25.34±1.31	25.31±1.26	0.35
	D_min_	0.40±0.15	0.40±0.16	0.35

Data presented as mean±standard deviation. Two-tailed *P* values from paired t tests. Abbreviation: D_max_ =  maximum dose; D_mean_ =  mean dose; D_min_ =  minimum dose.

HPC sparing plans generally showed an increase in the dose to the optic nerves and cochleae. Compared with non-HPC sparing plans, the average of D_mean_ and D_min_ to the optic nerves increased from 24.03 Gy and 6.51 Gy to 24.29 Gy and 6.76 Gy in HPC sparing plans, representing 1.1% and 3.8%, increase respectively (p<0.05). The mean dose for the cochleae also increased for the HPC sparing plans from 42.89 Gy to 43.39 Gy (p<0.05).

### Dose to hippocampus

The isodose distribution at the level of the hippocampi for one sample patient is shown in Figure 1C (non-HPC sparing IMRT plan) and Figure 1D (HPC sparing IMRT plan). For all plans, the mean hippocampal DVH parameters are listed in Table 5. Compared with non-HPC sparing plans, the mean D_max_, D_2%_, D_40%_, D_mean_, D_median_, D_98%_ and D_min_ for the HPC in the HPC sparing plans decreased by 29.2% (54.34 Gy vs 38.45 Gy), 33.1% (47.62 Gy vs 31.85 Gy), 49.0% (27.14 Gy vs 13.83 Gy), 41.4% (24.11 Gy vs 14.14 Gy), 41.7% (21.43 Gy vs 12.49 Gy), 13.5% (7.85 Gy vs 6.79 Gy) and 17.3% (6.94 Gy vs 5.74 Gy), respectively. The mean HPC V5, V10, V15, V20, V30, V40 and V50 in the non-HPC sparing plans were 99.64%, 89.45%, 65.5%, 52.81%, 32.39%, 12.16% and 2.81%, compared to 98.74%, 71.43%, 33.73%, 15.56%, 3.9%, 0.44% and 0.01% observed in the HPC-sparing IMRT. The V20 was reduced by as much as 37.25% on average in the HPC-sparing IMRT. Both dose and volume parameters for the HPC were significantly higher in the non-HPC sparing plans (p<0.05), except for D_min_ (P = 0.06) and V5 (P = 0.12).

**Table 5:** Hippocampal dosimetric and volumetric parameters for different IMRT plans.

Parameters	Non-sparing	Sparing	*P*-value
**D_max_ (Gy)**	54.34±6.83	38.45±8.61	0.00
**D_2%_ (Gy)**	47.62±8.00	31.85±4.98	0.00
**D_40%_ (Gy)**	27.14±5.44	13.83±1.71	0.00
**D_mean_ (Gy)**	24.11±2.39	14.14±1.59	0.00
**D_median_ (Gy)**	21.43±3.86	12.49±1.31	0.00
**D_98%_ (Gy)**	7.85±2.00	6.79±1.25	0.04
**D_min_ (Gy)**	6.94±2.10	5.74±1.34	0.06
**V_5_ (%)**	99.64±0.54	98.74±1.84	0.12
**V_10_ (%)**	89.45±8.36	71.43±12.15	0.00
**V_15_ (%)**	65.50±3.02	33.73±16.20	0.00
**V_20_ (%)**	52.81±6.87	15.56±6.79	0.00
**V_30_ (%)**	32.39±12.40	3.90±2.43	0.00
**V_40_ (%)**	12.16±6.70	0.44±0.80	0.00
**V_50_ (%)**	2.81±2.86	0.01±0.04	0.02

Data presented as mean±standard deviation. Two-tailed *P* values from paired t tests. Abbreviation: D_max_ =  maximum dose; D_mean_ =  mean dose; D_median_ =  median dose; D_min_ =  minimum dose; D_2%_ =  the dose delivered to 2% of the target volume; D_40%_ =  the dose delivered to 40% of the target volume; D_98%_ =  the dose delivered to 98% of the target volume; SD =  standard deviation. Vn =  percentage of volume receiving ≥n Gy.

## Discussion

With increasing long-term survival for patients with NPC treated with radiotherapy (RT), the late effects of RT have become more and more apparent. Cognitive dysfunction has been recognized as one such complication and has attracted much recent concern. Some studies [17], [18] have indicated that radiation-induced temporal lobe injury (RITLI) may be the source of cognitive function decline. Furthermore, a recent study [19] has suggested specifically that radiation-induced hippocampal damage plays a considerable role. However, the pathogenesis of radiation-induced cognitive dysfunction, and the correlation between the volume and location of RITLI and cognitive dysfunction have not been defined. One possible mechanism for radiation induced cognitive dysfunction is through the depletion of neural stem cells (NSC). The brains of all mammals, harbor a population of pluripotent NSC, some of which are located in the hippocampus and are involved in learning behaviors and memory [2]–[4]. Unlike other parts of the brain, these cells are readily induced to undergo apoptosis when irradiated and are therefore exquisitely sensitive to damage from radiation [10]. Radiation to the hippocampus, therefore, can deplete this population of NSCs through inducible apoptosis and subsequently result in cognitive decline.

Although the HPC plays an important role in late neurocognitive dysfunction, there has been no specification for HPC dose limitations in recent IMRT for NPC protocols such as RTOG 0225 and RTOG 0615. Similarly, the majority of clinical IMRT plans for NPC do not identify the HPC as an OAR. Therefore, the feasibility of HPC avoidance in NPC was previously unclear. To our knowledge, the present study is the first time that HPC sparing IMRT has been described for patients with NPC. In this dosimetric study, we were able to decrease hippocampal dose without affecting target coverage or homogeneity, while maintaining other OAR restrictions. The maximum and median dose to the hippocampus in our series of non-HPC sparing plans (54.34±6.83 Gy and 24.11±2.39 Gy) is in agreement with the conclusions of other authors [11], [20]. In our HPC sparing plans the maximum and median HPC doses decreased by 29% and 41%, respectively. Other dosimetric and volumetric parameters (D_2%_, D_40%_, D_median_, D_98%_, V_10_, V_15_, V_20_, V_30_, V_40_ and V_50_) also significantly decreased. Although comparative OAR doses were similar and achieved protocol dose criteria, the HPC sparing plans did show an increase in some dosimetric parameters for certain OARs. This was particularly true for low dose exposure to the optic nerves, brain-stem and cochleae with unclear clinical significance.

The exact radiation dose-volume relationship between the HPC and long-term cognitive dysfunction remains unclear. The results of two recent clinical experiments could shed some light. The preliminary results of RTOG 0933 showed that HPC sparing whole brain radiotherapy (WBRT) for brain metastases was associated with memory and quality of life preservation at up to 6 months follow-up, when compared to a historical control [21]. In this trial, the HPC was constrained to D_max_<16 Gy and D_100%_ = D_min_<9 Gy. This study, however excluded patients with any brain lesion within 5 mm of the HPC. Another study by Gondi et al. found that a D_40%_ of the bilateral hippocampi of greater than 7.3 Gy was associated with long-term impairment in list-learning delayed recall after radiotherapy for benign or low-grade adult primary brain tumors [22]. In our trial, D_max_ was 38.5 Gy and D_40%_ = 13.8 Gy in the HPC sparing plans. Both of these values are higher than those reported/required in the aforementioned trials. However, our D_100%_ of 5.7 Gy is less than the specified D_min_ of 9 Gy in RTOG 0933.

There are some important differences between our study and these other two investigations that warrant consideration. The patients in RTOG 0933 were all treated with WBRT to a prescription dose of 30 Gy in 10 fractions. In the current study, on the other hand, the patients treated for locally advanced NPC and a prescribed PTV dose of 59.4 Gy was required. In addition, the PTV was unavoidably adjacent to the HPC, making it extremely difficult and not feasible to meet the RTOG 0933 specified D_max_<16 Gy, while maintaining acceptable PTV coverage and avoidance of other OARs. It is also important to note the presence and importance of the dose-time factor (DTF) in determining normal tissue dose tolerances. RTOG 0933 required restriction of HPC D_max_ to less than 16 Gy (17 Gy acceptable). This 16 to 17 Gy, however, was given in 10 fractions, yielding ≤1.7 Gy per fraction. The mean D_max_ in our study was approximately 38 Gy primarily due to the high prescription dose; however, this was given in 33 fractions, meaning approximately 1.15 Gy per fraction, theoretically increasing CNS tissue tolerance. In the study by Gondi et al, the treated diseases, tumor locations, and target doses all differed significantly from our patients. This study was also limited by the small number of patients and the findings are yet to be confirmed by others. Taking these factors into account speaks to the paucity of data regarding the radiation tolerance of the HPC and begs the question if it is even appropriate to directly compare or extrapolate results from one series to another given the disparate patient populations, treated diseases and radiation dose and dose fractionation schema utilized.

Without knowledge of the ideal HPC dose constraints, we attempted to reduce HPC dose to the minimum, while maintaining sufficient PTV coverage and other OARs constraints. In this objective, we were able to statistically significantly reduce HPC dose while maintaining otherwise acceptable clinical treatment plans. As always, however, there remains the need for further research. Potential areas of investigation include newer techniques such as helical Tomotherapy or volume modulated arc therapy (VMAT) that could potentially further reduce the mean dose to the hippocampus in NPC irradiation, when compared with traditional IMRT [23]. Taheri-Kadkhoda et al [24] have also posited that using three-field intensity-modulated proton therapy (IMPT) has greater potential than 9-field IMRT with respect to tumor coverage and reduction of OAR integral dose (without HPC sparing) in RT of NPC patients. Another approach by Lin et al [25] is to treat NPC with reduced target volumes and to a lower prescription dose of 54–56 Gy for PTV2. In their study, this provided local control, disease-free survival and overall survival rates of 95%, 88%, and 90%, respectively, at 30 months of follow-up. Finally, certain drugs such as memantine and donepezil have been found to be neuroprotective for patients undergoing cranial radiation [26], [27], and the applicability of such therapies to NPC patients undergoing radiation should be investigated.

## Conclusions

Hippocampal sparing IMRT for nasopharyngeal carcinoma is feasible using a 9 field step-and-shoot approach. This strategy showed no significant differences in therapeutic target dosimetry and only minor changes in the dosimetry of other critical normal structures. Hippocampal sparing should be considered in IMRT treatment planning for locally advanced NPC in light of its feasibility, theoretical advantages in preserving cognitive function and lack of significant adverse impact on other dosimetric parameters.
